# Comparative Evaluation of Visual Observation, Digital Camera, and Smartphone Photography for Tooth Shade Selection: An In Vitro Study

**DOI:** 10.7759/cureus.105511

**Published:** 2026-03-19

**Authors:** Krithika Jothisankar, Reena Mittal, Ravi Madan, Mouli Sardar, Nishant Sinha, Sumaiya Iman, Marzina Rahman, Nandhu Krishna H, Seema Gupta

**Affiliations:** 1 Department of Prosthodontics, Kothiwal Dental College and Research Centre, Moradabad, IND; 2 Department of Orthodontics, Kothiwal Dental College and Research Centre, Moradabad, IND

**Keywords:** color, digital camera, matching, prosthodontics, smartphone, visual

## Abstract

Background: Accurate shade selection is critical for achieving esthetic success in fixed prosthodontics. This in vitro study aimed to compare the accuracy and reliability of shade selection using conventional visual assessments, digital camera photography, and smartphone photography.

Materials and methods: Ten shade tabs were evaluated by 10 observers (three professors, four postgraduate students, and three laboratory technicians), resulting in 100 observations per method. Shade matching was performed using the VITAPAN Classical shade guide (VITA Zahnfabrik H. Rauter GmbH and Co. KG, Bad Säckingen, Germany) under standardized daylight conditions (5000-6500 K). Visual selection relied on direct comparison, while digital camera and smartphone images were captured with consistent settings. Accuracy was determined based on agreement with a predetermined reference shade. Statistical analyses included Cochran’s Q test for overall differences, pairwise McNemar tests with Bonferroni adjustment, Fleiss’ kappa for inter-observer agreement, and Cohen’s kappa for intra-observer agreement between methods.

Results: Both digital camera and smartphone methods demonstrated significantly higher accuracy and consistency than the visual approach. Digital camera and smartphone photography showed substantial inter-observer reliability and strong agreement between the two digital techniques, effectively reducing the variability associated with observer experience. No meaningful differences were observed between the digital camera and smartphone methods.

Conclusion: Digital cameras and smartphone photography provided reliable, objective alternatives to conventional visual shade selection, offering improved accuracy and reproducibility. These techniques hold promise for enhancing shade communication in clinical prosthodontics, particularly in settings where accessibility and standardization are priorities. Further in vivo investigations are warranted to confirm its applicability in clinical conditions.

## Introduction

Esthetics play a pivotal role in contemporary prosthodontic practice, particularly in anterior restorations, where optimal color matching significantly influences treatment success and patient satisfaction. Among the various determinants of esthetic outcomes, accurate tooth shade selection remains one of the most challenging and technique-sensitive procedures [1]. An incorrect shade match may compromise the overall harmony of the restoration, leading to patient dissatisfaction despite technically sound treatment.

Conventionally, shade selection has been performed by visual comparison with commercially available shade guides, such as the VITAPAN Classical system (VITA Zahnfabrik H. Rauter GmbH and Co. KG, Bad Säckingen, Germany). Although widely practiced, owing to its simplicity and cost-effectiveness, the visual method is inherently subjective. It is influenced by multiple factors, including the observer’s age, sex, experience, visual acuity, color perception, and eye fatigue. Environmental factors such as lighting conditions, background, metamerism, and surrounding colors further affect shade determination. Even with normal color vision, human perception varies, thereby reducing reproducibility and reliability [2,3].

To overcome these limitations, digital methods, such as the use of intraoral scanners, digital spectrophotometry, and instrumental and photography-based methods, have been introduced [2-4]. Digital cameras combined with imaging software allow objective color analysis by quantifying color parameters. They enhance communication between clinicians and laboratory technicians and provide documentation for future reference [2,5]. Recently, smartphone cameras equipped with advanced imaging sensors and processing capabilities have gained popularity owing to their accessibility, portability, and ease of wireless data transmission [6,7]. However, despite their widespread use, there is limited evidence comparing their accuracy with that of conventional visual and digital camera methods under standardized conditions.

Therefore, this study aimed to comparatively evaluate the accuracy of visual, digital camera, and smartphone photography methods for shade selection in esthetic dentistry. The objectives were to assess the accuracy of visual shade matching, evaluate the effectiveness of digital photography using a digital camera, examine the accuracy of smartphone photography in shade determination, and compare the performance of all three methods in achieving accurate shade selection.

## Materials and methods

Study design and setting

This in vitro comparative study was conducted at the Department of Prosthodontics, Kothiwal Dental College and Research Centre, Moradabad, Uttar Pradesh from January 2025 to September 2025. This study was designed to compare the accuracy of three different shade selection methods: visual shade matching, digital camera photography-assisted shade matching, and smartphone photography-assisted shade matching under standardized conditions. Ethical approval was obtained from the Institutional Ethical Review Board (KDCRC/IERB/02/2024/34 dated 21.02.2024) prior to the commencement of the study. Written informed consent was obtained from all observers to participate in the study.

Sample size

Sample size calculation was performed using G*Power software (version 3.9.1, Heinrich Heine University Düsseldorf, Germany), considering the primary outcome as the difference in accuracy between the visual and digital shade selection methods, estimated at 28%. With a two-tailed alpha level of 0.05, 80% statistical power, and an anticipated absolute difference of 28% between paired proportions (McNemar’s test), the required minimum sample size was determined to be 100 paired observations. The study included 100 observations per method obtained from 10 observers who evaluated 10 shade tabs.

Observers’ selection and standardization

A total of 10 observers participated in the study, which included three professors, four postgraduate students, and three dental laboratory technicians. All observers were screened for normal color vision using Ishihara’s test for color deficiency (38-Plate Edition; Kanehara Trading Inc., Tokyo, Japan) [8]. The test was conducted under natural daylight conditions following the manufacturer’s instructions. Only observers who made fewer than five errors were included in the study to eliminate the influence of color vision deficiency. Observers were instructed regarding the study protocol and allowed practice sessions prior to data collection to minimize procedural bias.

Armamentarium

Two sets of VITAPAN Classical shade guides were used for shade matching procedures. A digital single-lens reflex camera (Canon EOS 600D, Canon Inc., Tokyo, Japan) was used to capture digital photographic images. A smartphone (iPhone 15, Apple Inc., Cupertino, CA, USA) was used for photographic imaging.

Image processing and shade guide preparation were performed using Adobe Photoshop CS3 Extended Version 10 (Adobe Systems Inc., San Jose, CA, USA) installed on a laptop with Windows 10 Pro operating system (Microsoft Corporation, Redmond, WA, USA), 8 GB RAM, and 238 GB storage capacity.

An 18% gray card (Neewer Technology Co., Shenzhen, China) was used to standardize the exposure and neutral-color balance. A tripod stand was used to mount the DSLR camera and a selfie stand was used to mount the smartphone. A Nissin Typodont Jaw Set - PRO2001-UL (Nissin Dental Products Inc., Kyoto, Japan) was used to prepare the concealed digital shade guides. Micropore tape (3M, Maplewood, MN, USA) was used to mask the identification numbers of shade tabs. Ishihara’s tests for color Deficiency, 38-Plate Edition, were used to assess color vision.

Sample selection

Ten observers, comprising three professors, four postgraduate students, and three dental technicians, were selected based on their clinical experience and willingness to participate. The observers provided informed consent before their inclusion in the study. Each observer performed shade matching using three methods: visual, digital photography, and smartphone photography. Each observer matched 10 shade tabs per method, resulting in 100 observations per method for a total of 300 observations.

Assessment of color vision

All observers were screened for color vision deficiency using Ishihara’s test (38-Plate Edition). The test was performed under natural daylight conditions to prevent color distortion. Each plate was presented at a distance of approximately 70 cm from the observer and was positioned perpendicular to the line of sight. The observers were instructed to identify the number within five seconds for each plate.

The first 17 plates were used for screening. Observers who made more than five errors were considered color-deficient and excluded from the study. Only those who correctly identified the screening plates and demonstrated normal color perception were included. The test was conducted individually in the presence of an investigator to maintain confidentiality and prevent external influences.

Preparation of visual shade guides

**Figure 1:**
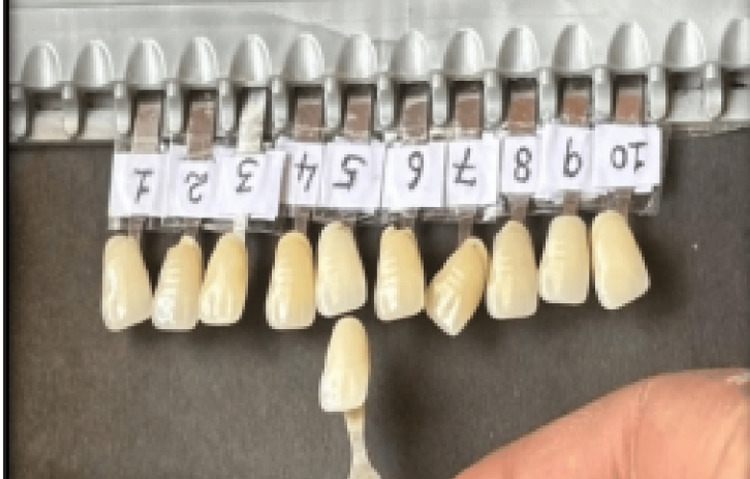
Visual shade matching procedure. Concealed VITAPAN Classical shade tabs with masked identification numbers arranged randomly for comparison with the control shade guide during visual shade matching under natural daylight conditions. Observers matched each concealed tab with the corresponding reference shade.

Two sets of VITAPAN Classical shade guides were used in this study. Ten shade tabs (A2, A3, A3.5, A4, B2, B3, B4, C2, C3, and D3) were selected for this study. One set served as the control shade guide, in which the identification numbers were kept visible. The second set served as the concealed shade guide, where identification numbers were masked using micropore tape and randomly labeled from 1 to 10. The concealed shade tabs were presented individually in a random sequence, and observers were instructed to match each concealed tab with the corresponding tab in the control shade guide (Figure 1).

Preparation of digital and smartphone photographic shade guides

For digital and smartphone shade guide preparation, the selected 10 shade tabs were placed over an 18% gray card at a standardized distance of 25 cm under northern daylight with a color temperature range of 5000-6500 K. For digital photography, a Canon EOS 600D camera was mounted on a tripod at a height of two feet. The standardized camera settings were maintained at ISO 200, shutter speed 1/125 s, aperture F11, focal length 55 mm, manual exposure mode, and no flash. Images were captured in the JPEG format with a resolution of 5184 × 3456 pixels at 72 dpi.

For smartphone photography, the iPhone 15 was mounted on a selfie stand at a height of 2 feet. Standardized settings included ISO 200, shutter speed of 1/50 s, aperture of f/1.6, and focal length of 26 mm. Images were captured in the JPEG format with a resolution of 6048 × 8064 pixels under similar daylight conditions. The reported resolution corresponds to the native image output of the device. Image orientation did not influence shade evaluation because the shade tabs were digitally isolated and standardized during image processing prior to observer assessment. All images were transferred to a laptop and processed using the Adobe Photoshop CS3 Extended software. The shade tabs were isolated using a pen tool and layered against a black background to create digital control shade guides.

**Figure 2:**
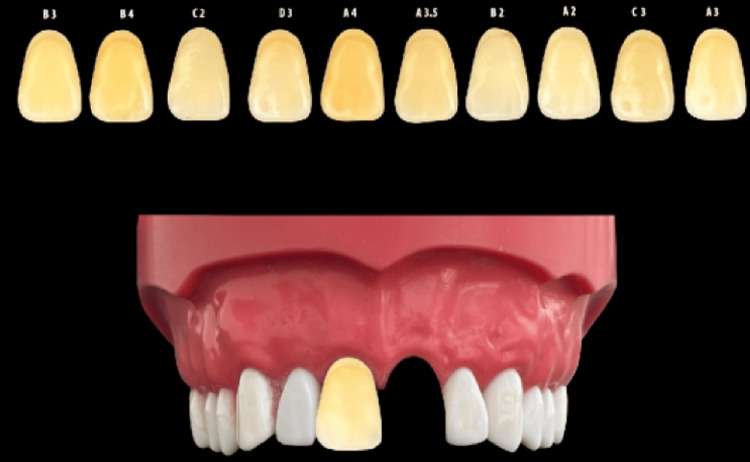
Digital image-based shade selection. Representative digital setup showing the typodont with maxillary anterior teeth and digitally isolated shade tabs used for photographic shade matching. The concealed shade tab was positioned in the right central incisor region and compared with digitally prepared control shade tabs using image-processing software.

For concealed digital shade-guide preparation, a photograph of the typodont with six clearly visible maxillary anterior teeth was captured under identical standardized settings. The image was layered and the relevant area was selected using a pen tool. Image segmentation was performed by a single trained investigator following a predefined editing protocol using identical zoom levels and canvas settings. The pen tool was used only to isolate the shade tab boundaries, and no adjustments were made to hue, saturation, brightness, or color balance to preserve the original color information. Each concealed shade tab with a masked identification number was digitally positioned in the right central incisor region. Ten separate Photoshop files were created for each concealed shade tab, with all the control shade tabs positioned for comparison (Figure 2).

Procedure for shade matching

Visual shade matching was conducted between 9 a.m. and 10 a.m. under natural northern daylight at a room temperature of approximately 26^0^C. All observations and photographic procedures were performed during the same time interval (9:00-10:00 a.m.) to minimize variations in natural daylight. An 18% grey card was used for color calibration and identical camera parameters, positioning, and background conditions were maintained for all sessions to ensure consistency in illumination conditions. The control and concealed shade guides were arranged on a flat black background. The observers independently matched each concealed shade tab to a control shade guide. To prevent eye fatigue, observers were instructed to rest their eyes by closing them or viewing a gray card intermittently for 15 s. Selected shade tab numbers were recorded, and correct matches were counted. For digital and smartphone photographic shade matching, observers compared the concealed digital shade tab with digital control shade tabs displayed on the screen using the prepared Photoshop files. The numbers of correct and incorrect matches were recorded. Each observer completed 10 observations per method.

Data collection and statistical analysis

A total of 300 observations were recorded, comprising 100 observations for each shade-matching method. Observations were categorized as correct or incorrect based on matching with a predetermined reference shade. Statistical analyses were performed using IBM SPSS Statistics, version 26.0 (IBM Corp., Armonk, NY, USA). The accuracy of shade matching for the visual, digital camera, and smartphone methods was calculated as proportions with 95% confidence intervals. Differences in related proportions were assessed using Cochran’s Q test, followed by pairwise McNemar’s tests with Bonferroni adjustment for multiple comparisons. Interobserver agreement for each method was evaluated using Fleiss’ Kappa statistics, whereas intra-observer agreement between methods was assessed using Cohen’s kappa coefficient. Statistical significance was set at P < 0.05.

## Results

The study included 300 observations obtained from 10 observers (three professors, four postgraduate students, and three laboratory technicians) who evaluated 10 shade tabs using three different methods: conventional visual shade selection, digital photography with a dedicated camera, and digital photography using a smartphone. The overall accuracy of shade matching differed substantially between the three methods (Table 1). The conventional visual method resulted in 48 (48%) correct matches out of the 100 observations. In contrast, the digital camera method achieved 77 (77%) correct matches, whereas the smartphone method yielded 76 (76%) correct matches. The confidence intervals for both digital methods were clearly separated from and higher than those for the visual method, indicating superior performance.

**Table 1:** Overall accuracy of shade matching by each method. N = observations per method, Accuracy = (number of correct shade matches / total observations) × 100, Confidence intervals calculated using the Wilson score method.

Method	Correct N (%)	Incorrect N (%)	Total N	Accuracy	95% Confidence Interval
Visual	48 (48)	52 (52)	100	48%	42.1 – 55.9
Digital camera	77 (77)	23 (23)	100	77%	70.9 – 82.2
Smartphone	76 (76)	24 (23)	100	76%	69.8 – 81.4

Cochran’s Q test confirmed a highly significant overall difference in the proportion of correct shade matches among the three methods (Q = 94.62, df = 2, p < 0.001) (Table 2).

**Table 2:** Overall comparison of shade matching accuracy across methods – Cochran’s Q test. *Statistically significant at p < 0.05

Test	Q value	Degrees of freedom (df)	p-value
Cochran’s Q	94.62	2	0.001*

Subsequent pairwise post hoc comparisons using McNemar tests with Bonferroni adjustment (α = 0.016) demonstrated that both digital methods were significantly more accurate than the visual method. The digital camera method was associated with 5.31 times higher odds of a correct match compared to visual assessment (p = 0.001; 95% CI: 2.78-10.12) (Table 3).

**Table 3:** Pairwise comparison between digital camera and visual shade matching using McNemar test. Bonferroni adjusted α = 0.016, *Statistically significant at p < 0.05, CI: confidence interval, OR: odds ratio.

Variable	Digital camera correct	Digital camera incorrect	Total	Chi stats	p-value	OR (CI 95%)
Visual correct	41	7	48	34.09	0.001*	5.31 (2.78 – 10.12)
Visual incorrect	36	16	52			
Total	77	23	100			

Similarly, the smartphone method showed 4.86 times higher odds of correct shade selection than did the visual method (p = 0.001; 95% CI: 2.59-9.12) (Table 4).

**Table 4:** Pairwise comparison between smartphone and visual shade matching using McNemar test. Bonferroni adjusted α = 0.016, *Statistically significant at p < 0.05, CI: confidence interval, OR: odds ratio.

Variable	Smartphone correct	Smartphone incorrect	Total	Chi stats	p-value	OR (CI 95%)
Visual correct	42	7	48	31.85	0.001*	4.86 (2.59 – 9.12)
Visual incorrect	34	17	52			
Total	76	24	100			

No statistically significant difference was observed between the digital camera and smartphone methods (p = 0.70; odds ratio = 1.17; 95% CI: 0.52-2.63) (Table 5).

**Table 5:** Pairwise comparison between smartphone and digital camera shade matching using McNemar test. Bonferroni adjusted α = 0.016, *Statistically significant at p < 0.05, CI: confidence interval, OR: odds ratio.

Variable	Smartphone correct	Smartphone incorrect	Total	Chi stats	p-value	OR (CI 95%)
Digital camera correct	70	7	77	0.15	0.70	1.17 (0.52 – 2.63)
Digital camera incorrect	6	17	23			
Total	76	24	100			

Accuracy varied according to the observer's experience level (Table 6). Professors achieved the highest performance using the visual method, followed by postgraduate students and laboratory technicians. With the digital camera, the accuracy improved to 24/30 (80%) among professors, 31/40 (77%) among postgraduate students, and 22/30 (73%) among laboratory technicians. The smartphone-based assessment produced similar gains, reaching 25/30 (83%) for professors, 30/40 (75%) for postgraduate students, and 22/30 (73%) for laboratory technicians. These results indicate that, while professional experience was associated with better baseline performance, digital methods produced substantial and consistent improvements across all observer categories.

**Table 6:** Accuracy of shade matching by observer category and method. N (%): Number of observation (percentage), Total N: Total observations per method (number of observers (n) × 10 teeth), Percentages rounded to the nearest whole number.

Observer group	Method	Correct N (%)	Total N	Accuracy
Professors (n = 3)	Visual	17 (57)	30	57%
	Digital camera	24 (80)	30	80%
	Smartphone	25 (83)	30	83%
Postgraduate students (n = 4)	Visual	18 (45)	40	45%
	Digital camera	31 (77)	40	77%
	Smartphone	30 (75)	40	75%
Lab technicians (n = 3)	Visual	13 (43)	30	43%
	Digital camera	22 (73)	30	73%
	Smartphone	22 (73)	30	73%

Inter-observer agreement, assessed using Fleiss’ kappa, was moderate for the visual method (κ = 0.42), reflecting considerable variability among observers. Agreement improved markedly to the substantial range with both digital techniques: κ = 0.68 for the digital camera and κ = 0.66 for the smartphone method (Figure 1). This pattern suggests that digital shade selection reduces subjective differences and enhances the consistency among observers.

**Figure 3:**
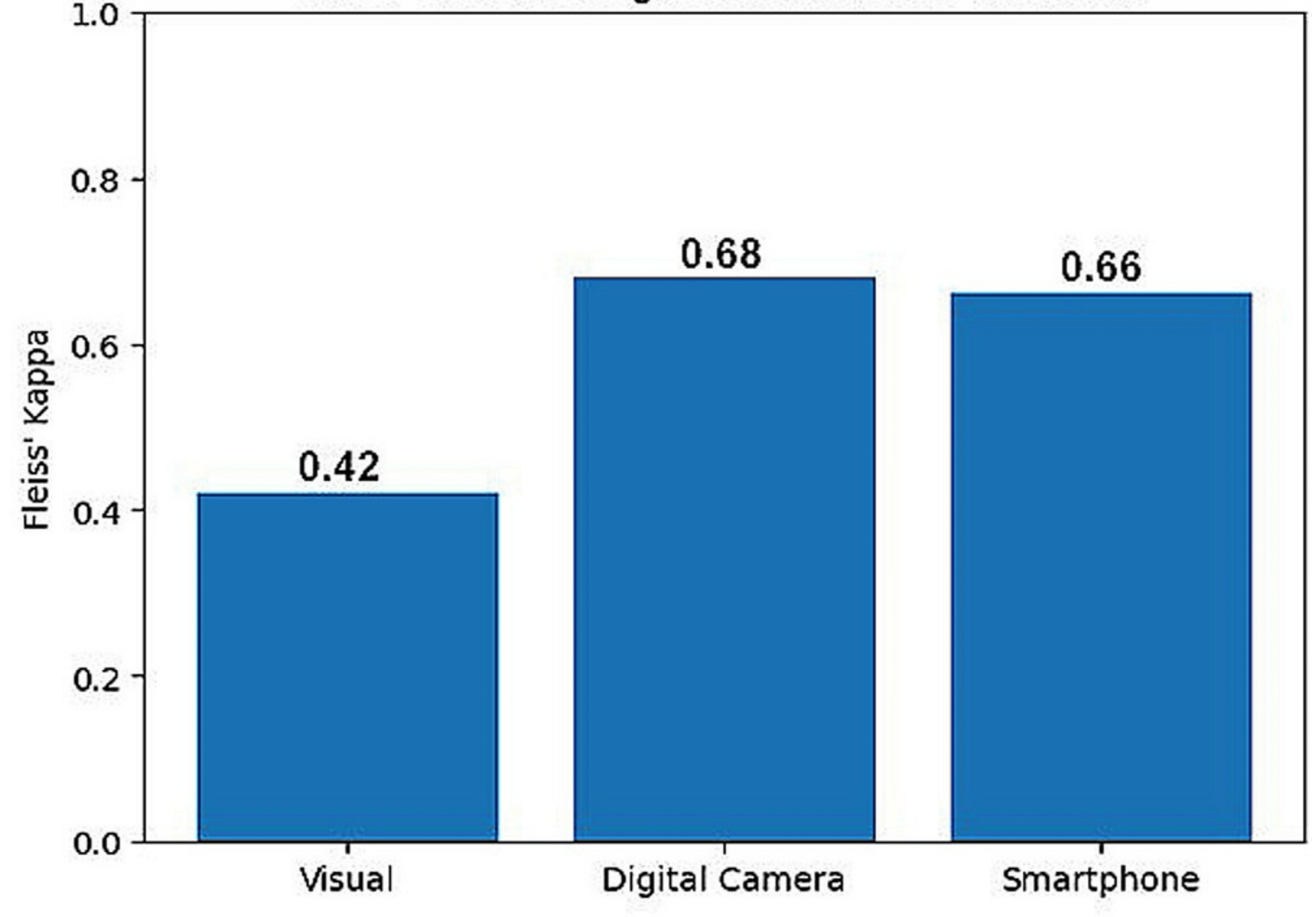
Inter-observer agreement across shade matching methods (Fleiss’ kappa).

**Figure 4:**
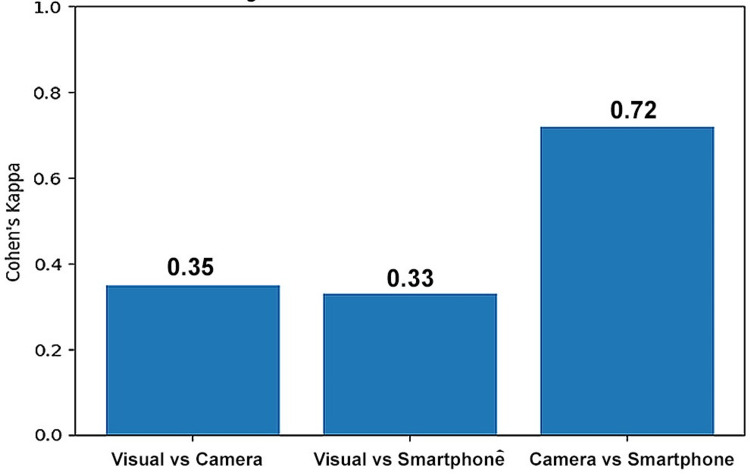
Pairwise intra-observer agreement between shade matching methods (Cohen’s kappa).

Intra-observer agreement between methods, evaluated using Cohen’s kappa, was only fair when comparing visual assessment with either digital technique (visual vs. digital camera: κ = 0.35; visual vs. smartphone: κ = 0.33) (Figure 4). In contrast, the agreement between the two digital methods was substantial (κ = 0.72), indicating high reproducibility when the same observer used camera-based versus smartphone-based digital capture.

In summary, both digital shade selection methods demonstrated significantly higher accuracy, superior inter-observer reliability, and greater between-method consistency than conventional visual shade matching. The performances of dedicated digital cameras and smartphones were statistically equivalent, suggesting that smartphone-based shade selection represents a reliable and accessible alternative in clinical and laboratory settings.

## Discussion

The results of the present study demonstrated that both digital methods significantly outperformed the visual approach, with the digital camera achieving 77% accuracy and smartphone photography 76%, compared with 48% for visual matching. The overall difference among the methods was statistically significant, highlighting the superior reliability of the technology-assisted techniques. Pairwise comparisons confirmed that the digital camera and smartphone methods yielded higher correct matches than visual assessment, while no significant difference existed between the two digital approaches. These findings underscore the potential of digital tools to mitigate subjectivity in shade selection.

The enhanced accuracy of the digital and smartphone methods aligns with several studies that emphasize the limitations of visual shade matching. Visual determination is inherently subjective and influenced by observer experience, lighting conditions, and metamerism, where colors appear different under varying light sources [9-11]. By contrast, digital photography allows for standardized image capture and analysis using software such as Adobe Photoshop, enabling precise color comparison in a controlled environment [11,12]. For instance, Schropp reported that digital photographs analyzed using software were more accurate than visual methods because of reduced perceptual variability [11]. Similarly, Jarad et al. found that individual observer performance improved significantly with computerized matching [12]. Kelkar et al. noted that digital photography, especially with polarizing filters, provides better results than visual assessment [13]. The current study's use of a gray card and daylight (5000-6500 K) minimized lighting discrepancies and supported consistent outcomes.

Smartphone photography has emerged as a comparable alternative to dedicated digital cameras, with no significant differences in accuracy. This is consistent with Mohammadi et al., who showed high validity and reliability of smartphone images analyzed using Adobe Photoshop compared to spectrophotometers [6]. Alsahafi et al. reported superior accuracy in hue, value, and chroma using smartphones compared to visual and instrumental methods [14]. Tam and Lee demonstrated feasible shade classification using smartphone cameras and machine learning, achieving high accuracy [15]. Rondon et al. found photographic analysis to be more precise than visual matching under standardized protocols [16]. The accessibility of smartphones makes them a cost-effective tool for clinical use, as noted by Abraham et al. for grayscale value determination [17]. In the present study, the slight edge of digital cameras may stem from a higher resolution (5184 × 3456 pixels vs. 6048 × 8064, but optimized settings), yet the ubiquity of smartphones offers practical advantages.

Observer experience influenced baseline performance, with professors achieving higher visual accuracy than students and technicians, corroborating Paul et al.'s findings on the consistency of experienced observers [18]. However, digital methods have reduced this gap, improving accuracy across all groups, as seen in Alshiddi et al.'s comparison of trained and untrained participants [19]. Udiljak et al. highlighted technicians' superior precision owing to their routine fabrication tasks [20]. The inclusion of diverse observers in this study reflects real-world variability, but digital tools democratize accuracy and minimize experience-related bias.

Clinically, these findings imply that integrating digital and smartphone photography into prosthodontic workflows can enhance shade communication between clinicians and laboratories, thereby reducing remakes and patient dissatisfaction. Smartphone methods, being portable and low-cost, are particularly useful in resource-limited settings or teledentistry. They facilitate objective documentation of treatment planning and legal records. Adopting standardized protocols such as zero-contact distance, perpendicular angulation, and daylight conditions can optimize the results. This could improve esthetic outcomes in fixed prostheses, veneers, and crowns, where shade mismatch affects patient satisfaction.

Limitations include the in vitro design and use of artificial setups that do not replicate intraoral conditions such as moisture, angulation, or adjacent teeth influence. The VITAPAN Classical guide has limited shades, and the VITA 3D-Master offers more options. No time limit was imposed, potentially overlooking eye fatigue. Experience was categorized but not quantified, and the small sample (300 observations) limited generalizability. Another limitation of the study is that the photographic methods did not specifically account for the principle of metamerism, where colors may appear different under varying lighting conditions. Additionally, polarizing filters were not used during image capture to reduce surface reflections or glare, which may have introduced minor variations in color perception. Future studies should incorporate in vivo scenarios, larger cohorts, advanced software based on artificial intelligence, and multiple shade guides under varied lighting.

## Conclusions

Within the constraints of this in vitro investigation, both digital camera photography and smartphone-based photography proved to be markedly superior to conventional visual shade selection, in terms of accuracy and consistency. Digital approaches demonstrated significantly higher reliability and reduced inter-observer variability compared to the subjective nature of visual matching. Moreover, the two digital methods exhibited equivalent performance, with substantial agreement between them, and improved reproducibility across observers with varying experience levels. These outcomes highlight the value of objective technology-assisted techniques in overcoming the inherent limitations of human visual perception during shade determination. Adopting standardized digital photography protocols in clinical prosthodontic practice has the potential to enhance shade communication with dental laboratories, minimize remakes, and improve overall esthetic predictability and patient satisfaction. Future in vivo studies under diverse clinical conditions are recommended to substantiate these findings further and facilitate broader implementation.
